# Zebrafish neuromesodermal progenitors undergo a critical state transition *in vivo*

**DOI:** 10.1016/j.isci.2022.105216

**Published:** 2022-09-26

**Authors:** Kane Toh, Dillan Saunders, Berta Verd, Benjamin Steventon

**Affiliations:** 1 Department of Genetics, University of Cambridge, Cambridge CB2 3EH, UK; 2 Department of Zoology, University of Oxford, Oxford OX1 3SZ, UK

**Keywords:** Biological sciences, Developmental biology, Bioinformatics

## Abstract

The transition state model of cell differentiation proposes that a transient window of gene expression stochasticity precedes entry into a differentiated state. Here, we assess this theoretical model in zebrafish neuromesodermal progenitors (NMps) *in vivo* during late somitogenesis stages. We observed an increase in gene expression variability at the 24 somite stage (24ss) before their differentiation into spinal cord and paraxial mesoderm. Analysis of a published 18ss scRNA-seq dataset showed that the NMp population is noisier than its derivatives. By building *in silico* composite gene expression maps from image data, we assigned an ‘NM index’ to *in silico* NMps based on the expression of neural and mesodermal markers and demonstrated that cell population heterogeneity peaked at 24ss. Further examination revealed cells with gene expression profiles incongruent with their prospective fate. Taken together, our work supports the transition state model within an endogenous cell fate decision making event.

## Highlights

• Gene expression variability of zebrafish NMps peaks at the 24 somite stage (24ss).
• The critical transition index peaks in 18ss NMps relative to their derivatives
• *In silico* gene expression composite maps were reconstructed from HCR confocal images
• ‘Rebellious’ cells with incongruent state to fate relationship increased at 24ss

## Graphical abstract

**Figure:**
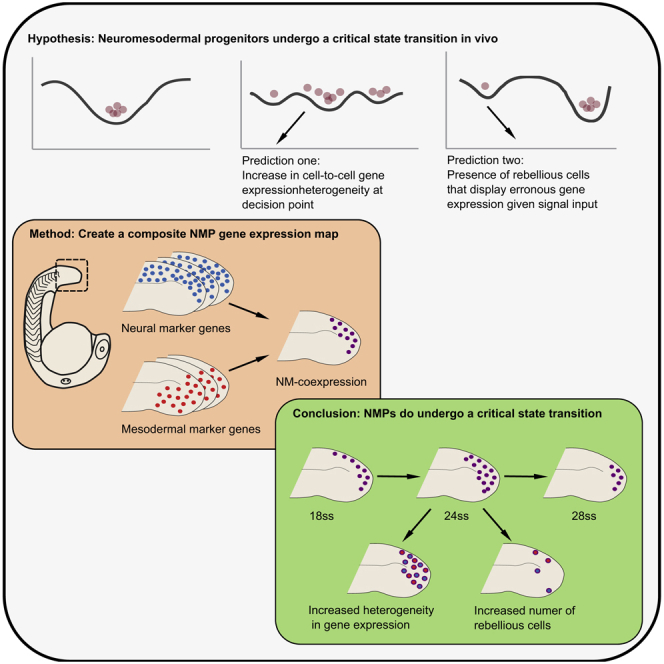

## Introduction

Neuromesodermal progenitors (NMps) are axial progenitors that co-express the lineage-specific transcription factors *Brachyury/T/Tbxta* and *Sox2* and are competent to generate both neural (e.g. spinal cord) and mesodermal (e.g., somite) fates at the single-cell level (Henrique et al., 2015; Wymeersch et al., 2021). Bipotent NM cells have been identified in amniotes such as mouse (Cambray & Wilson, 2002, 2007) and chick (Brown and Storey, 2000; Guillot et al., 2021; Wood et al., 2019) as well as anamniotes such as *Xenopus* (Davis and Kirschner, 2000; Gont et al., 1993), axolotl (Taniguchi et al., 2017) and zebrafish (Martin and Kimelman, 2012). Therefore, they are an evolutionary conserved cell population whose decision to generate spinal cord and paraxial mesoderm provides an ideal system to explore the mechanisms of cell fate decision making *in vivo*.

Sox2 is a member of the family of B1 Sox transcription factors, which in zebrafish also includes Sox1a/1b/3/19a/19b (Hu et al., 2021). These B1 transcription factors are functionally redundant, and quadruple knockdown of Sox2/3/19a/19b demonstrated that together with Pou5f1 (Oct4) or Otx2, they play critical roles in neural differentiation by regulating proneural genes (Neurog1, Her3) and signaling pathway genes (Cyp26a1, Shh) (Okuda et al., 2010). Tbxta is an ortholog of Brachyury, which is a T box transcription factor that directs posterior mesoderm formation (Martin and Kimelman, 2008; Schulte-Merker et al., 1994). Chromatin immunoprecipitation experiments have identified the direct regulatory targets of Tbxta (Tbx16, Eve1, Fgf8, Sp5l), demonstrating that Tbxta is the key orchestrator of posterior mesoderm formation (Morley et al., 2009). Thus, Sox2 and Tbxta can be considered as primary regulators of the neural and mesodermal program respectively.

The degree to which NMps divide to produce daughter cells of both neural and mesodermal fates depends on species-specific growth dynamics (Steventon and Martinez-Arias, 2017). In the zebrafish embryo, there is little volumetric growth associated with posterior body elongation (Steventon et al., 2016) and proliferation stops abruptly within the embryo around the 10 somite stage (ss) (Bouldin et al., 2014; Zhang et al., 2008). Correspondingly, zebrafish tailbud NMps are a largely quiescent pool of mono-fated progenitors that give rise to a limited portion of the posterior body axis (Attardi et al., 2018; Bouldin et al., 2014). In the mouse embryo, using retrospective clonal analysis, long clones originating from a single cell have been observed in both neural and mesodermal tissues (Tzouanacou et al., 2009), which is consistent with a proliferative phase in the mouse NMps at around E9.5 (Wymeersch et al., 2016). Despite this difference in developmental dynamics, two independent lines of evidence support the notion that zebrafish NMps are, like all other vertebrate NMps, competent toward both neural and mesodermal fates. First, single cell transplantation experiments demonstrate that zebrafish NMps can be steered toward either neural or mesodermal fates on manipulation of the canonical Wnt pathway (Martin and Kimelman, 2012). Second, a single cell transcriptomic signature that contains conserved markers of both spinal cord and paraxial mesoderm states have been discovered for the zebrafish NMps at late gastrulation/early tailbud stages of development (Lukoseviciute et al., 2021). Thus, a conceptual clarification between NM competent cells and NM progenitors (NMps) has been proposed, of which a differing proportion of NM competent cells act as NMps in a stage- and species-specific manner dependent on the rate of proliferation (Binagui-Casas et al., 2021; Sambasivan and Steventon, 2021). In this article, we refer to these cells as zebrafish tailbud ‘NMps’ to remain consistent with previous literature, although they are better understood as NM competent cells at post 10ss of development.

How do these zebrafish tailbud NMps differentiate into their NM derivatives? Differentiation has been widely characterized as an ordered and largely deterministic succession of cellular states, specifically transcriptomic states, that emerges from the activation of a set of master transcription factors in a gene regulatory network (Davis et al., 1987; Whyte et al., 2013). In this article, we define “cell states” to refer to specifically to transcriptomic states. If transcriptomic states strongly correlate with developmental lineage, then we can sort single cells along a pseudotemporal axis of developmental progression using their transcriptomic states as the similarity measure and infer the gene expression trajectories within these differentiating cells. Elucidating the pseudotemporal axis has uncovered numerous insights into development (Wagner et al., 2018; Wolf et al., 2019) and disease (Mukherjee et al., 2020; Petti et al., 2022). Despite their utility, pseudotemporal ordering algorithms make a critical simplifying assumption: cells with similar transcriptomic profiles are assigned to be closer together in their developmental maturity along a lineage (Schier, 2020; Tritschler et al., 2019). This biological assumption has been challenged by several observations. First, *in vitro* studies revealed the prevalence of non-genetic heterogeneities within clonal stem cell populations, where cells stochastically transition between distinct metastable states despite being functionally homogeneous (Canham et al., 2010; Hayashi et al., 2008; Trott et al., 2012). In addition, global transcriptomic trajectories may be driven by complex dynamics such as slow fluctuations that persist across cell division cycles (Chang et al., 2008) and oscillatory dynamics in key regulators (Verd et al., 2018). Furthermore, distinct trajectories may converge to the same terminal fate (Packer et al., 2019). These observations suggest that the relationship between cell fate and transcriptomic state can be complex (Casey et al., 2020) and additional information is required before constraining the possible dynamics that arise from snapshot data with the maximum parsimony assumption (Tanay and Regev, 2018; Weinreb et al., 2018).

Given the prevalence of non-genetic heterogeneities, cellular differentiation models have been developed to account for their role in differentiation. Generally, these models involve two qualitatively distinct phases - an initial period of increased stochasticity where cells dynamically explore a broader region of state space followed by the convergence into cell-type specific gene expression profiles. During the initial stochastic phase, transcriptomic states and cell fates are less correlated as gene expression heterogeneity increases. Taking a statistical mechanical perspective, this phenomenon of ‘regulated stochasticity’ is consistent with cellular differentiation being a critical phase transition (Teschendorff and Feinberg, 2021). Models that belong in this class include the Darwinian model of cellular differentiation (Capp and Laforge, 2020; Laforge et al., 2005; Kupiec, 1997; Kupiec, 2014; Minelli and Pradeu, 2014; Paldi, 2020), the ‘exploratory’ model of stem cell decision-making (Halley et al., 2009) and the ‘transition state’ model (Antolović et al., 2019; Brackston et al., 2018; Moris et al., 2016; Martinez-Arias and Hayward, 2006; Muñoz-Descalzo et al., 2012; Rué and Martinez Arias, 2015).

Initial non-genetic heterogeneity models came from early studies of hematopoietic progenitors, which found that the progenitors simultaneously co-express genes from multiple lineages, producing a promiscuous gene expression profile in a phenomenon called multilineage priming. (Hu et al., 1997; Laslo et al., 2006; Mikkers and Frisen, 2005). This observation has also been observed *in vivo*. Cells of the early Xenopus gastrula were found to express genes from multiple germ layers, and when exposed to the mesendodermal-inducing factor Activin, single cells co-express both mesoderm and endoderm genes within the same cell (Wardle and Smith, 2004). Recent single-cell RNA sequencing (scRNA-seq) studies of *Caenorhabditis elegans* also noted the occurrence of multilineage priming in numerous lineage branches (Packer et al., 2019), providing further evidence against the strict deterministic, mosaic view of *C. elegans* development that dominated early thinking (Martinez-Arias et al., 2013).

Experimental observations of a surge in gene expression variability that precedes a commitment phase are found predominantly in *in vitro* models such as hematopoietic stem cell differentiation models (Dussiau et al., 2022; Hu et al., 1997; Mojtahedi et al., 2016; Moussy et al., 2017; Pina et al., 2012; Richard et al., 2016), induced pluripotent stem cells (iPSCs) (Bargaje et al., 2017; Buganim et al., 2012) and mouse embryonic stem cells (mESCs) (Moris et al., 2018; Semrau et al., 2017; Stumpf et al., 2017). In contrast, *in vivo* observations of this phenomenon have been comparatively rare (Antolović et al., 2019; Peláez et al., 2015). *In vivo* evidence are vital to ensure that the preceding *in vitro* observations are not due to artifacts of cell culture conditions (MacArthur and Lemischka, 2013; Smith, 2013) or reporter dynamics (Smith et al., 2017).

Several indices have been developed to quantify gene expression variability. In a study of blood progenitor cells, the critical transition index (I_c_) was developed and shown to gradually increase as cells approach the critical transition point. Ic is defined as a ratio of two averaged Pearson correlation coefficients: the average correlations between all pairs of gene vectors divided by the average correlations between all pairs of cell state vectors (Mojtahedi et al., 2016). Intuitively, as the cell population approaches the transition, the cell-cell correlation term in the denominator decreases as cells become dissimilar from one another and the gene-gene correlation term in the numerator increases as subset of genes change in concert, resulting in a surge in the index. The critical transition index is similar to another index called the I score, which is computed from a smaller set of dynamical network biomarkers (Chen et al., 2012,^2015^), in that they are both derived from dynamical systems theory. Another measure of gene expression variability, derived from information theory, is the Shannon entropy. Shannon entropy is defined for a probability distribution and measures the extent of departure from the uniform distribution - the flatter the distribution, the greater the entropy and the greater the degree of uncertainty (MacArthur and Lemischka, 2013). In particular, the Shannon entropy can be computed per cell (intracellular entropy) or per gene (intercellular entropy), where the latter aims to capture the gene expression variability of genes across an entire cellular population (Gandrillon et al., 2021). In our analysis of the NMp population, we compute both I_c_ and the intercellular entropy of the NM index distribution to examine the gene expression variability of the NM population. We compare different regions of the tailbud where progenitor cells are either in neural, mesodermal or NM states as a proxy for different timepoints of their differentiation.

In this article, we assessed the transition state hypothesis *in vivo* during the zebrafish tailbud NMp differentiation event. Our results can be grouped according to two features of the hypothesis:

### Transient increase in transcriptional heterogeneity during NMp differentiation

As photolabels of the NMp region at the 12ss revealed that cells only contribute to somites and spinal cord from the 24 somite level onwards (Attardi et al., 2018), we focused on a time-window between the 18 and 30ss to capture the commitment event. By quantifying the single-cell levels of nuclear *sox2* and *tbxta* expression in NMps from 18ss to 30ss*in situ,* we demonstrate that the heterogeneity in expression of both genes as well as the variability in NMp number peak at 24ss. In addition, by examining a publicly available 18ss scRNA-seq dataset of the zebrafish embryo (Wagner et al., 2018), we found that NMps have a higher critical index and transcriptional noise relative to their derivatives, supporting the view that the NMp population is noisier relative to their derivatives. Furthermore, by combining the expression of multiple NMp marker genes across multiple samples with an image alignment pipeline (ZebReg) and computing the ‘NM index’, we found that the intercellular Shannon entropy, a measure of the population heterogeneity, also peaks at 24ss.

### Loosening of the relationship between cell state and cell fate: Existence of ‘rebellious’ cells

We exploited the relative biological simplicity of the zebrafish NMp system to relate cellular states to cellular fates by examining the spatial locations of the NMps within our ZebReg composite maps. We identified an increase in the number of cells expressing a neural gene expression profile at the 24ss within the mesoderm-fated domain. These are cells possessing a transcriptomic profile that is incongruent with their prospective fate, indicative of an uncoupling of the relationship between cell state and cell fate. Following the work of Mojtahedi et al., we labeled these cells as ‘rebellious’.

Taken together, our work supports the existence of a transition state and the presence of ‘rebellious’ cells *in vivo* during zebrafish NMp differentiation.

## Results

### Heterogeneity in *sox2* and *tbxta* expression and variability in the number and locations of NMps peak at 24ss

**Figure 1:**
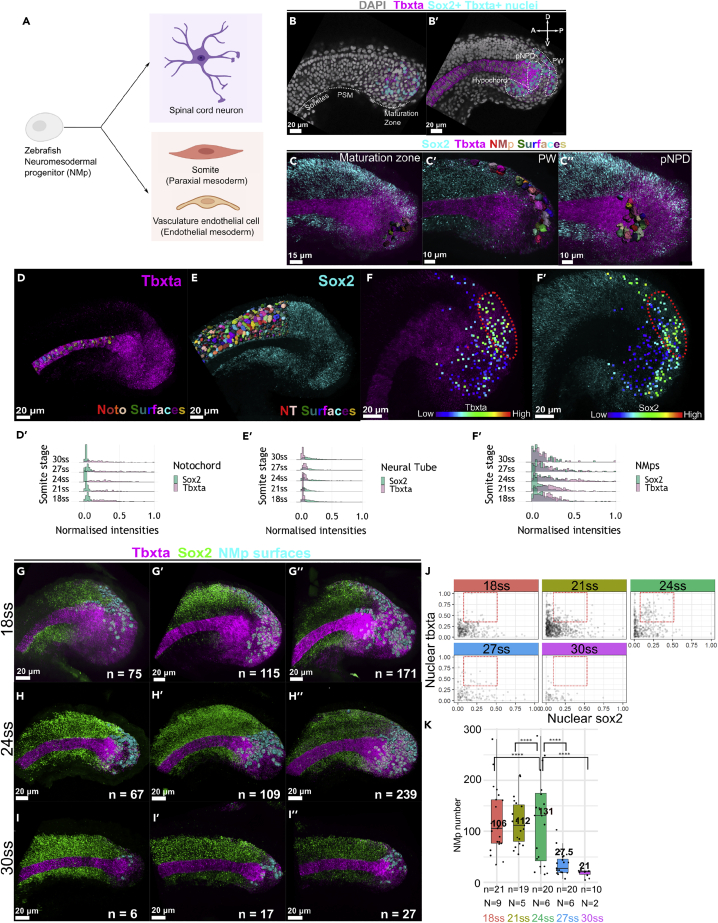
Heterogeneity in *sox2* and *tbxta* expression and variability in the number and locations of NMps peak at 24ss (A) Zebrafish NMps undertake a binary fate decision to differentiate into the posterior neural and mesodermal fates. (B) 2D lateral slice showing *sox2*+*tbxta*+ nuclei (cyan surfaces) in the maturation zone. (B′) 2D medial slice showing *sox2*+*tbxta*+ nuclei in the hypochord, pNPD and PW. pNPD: posterior notochord progenitor domain; PW: posterior wall. (C–C″) Segmented NMp surfaces located in the (C) maturation zone (C′) PW (C″) pNPD. (D, E, and F–F′) Maximum intensity projections of *tbxta* and *sox2* shown alongside segmented surfaces of the posterior notochord (D) and posterior neural tube (E). NMps are shown as points colored according to their (F) *tbxta* and (F′) *sox2* expression levels. The red regions highlight the NMps in the posterior wall that co-express intermediate levels of (F′) *tbxta* and (F″) *sox2*, which is also highlighted with a red region in (J). (D′, E′, and F″) Histograms from 18ss to 30ss depicting the expression distributions of nuclear sox2 and tbxta distributions in the (D′) posterior notochord (E′) posterior neural tube (F′) NMp populations. Each ridge plot displays the expression distributions of the specified cell population across all analyzed samples. All three cell populations within a sample adopted the same Sox2 and Tbxta threshold value for min-max normalization (STAR methods: Quantification and normalization of nuclear gene expression intensities in NMps). (G–I″) HCR-stained samples at (G-G″) 18ss, (H–H″) 24ss and (I–I″) 30ss with three representative images per set. n: number of segmented NMps in each sample. (J) Scatterplots of *sox2* and *tbxta* expression of NMps from 18ss to 30ssat three-somite intervals. Each point corresponds to the normalised nuclear *sox2* and *tbxta* intensities of a single NMp. The red boxes at each stage highlight NMps with intermediate levels of both genes. (K) Box and whisker plots of the number of NMps from 18ss to 30ssat three-somite intervals. Each point corresponds to the number of NMps in a single sample. The median NMp number is indicated in bold. n: total number of samples analyzed for each stage (biological replicate). N: number of distinct imaging experiments, where different biological samples imaged on the same day are considered a single imaging experiment. Levene’s test for the equality of variance was carried out for the NMp numbers at 24ss against the other four timepoints. ∗pvalue < 0.01.

To assess the number and location of zebrafish tailbud NMps over time, we performed HCR stains for *sox2* and *tbxta* to quantify the mRNA expression of single cells *in situ* within the zebrafish tailbud (Figures S1 and S2A–S2E). First, we compared the expression of nuclear *sox2* and *tbxta* in the NMps against the posterior notochord and posterior neural tube populations (Figures S2F–S2H′). We find that the posterior notochord population has a tight distribution of nuclear *sox2* with a mean normalized intensity close to 0 and a broader nuclear *tbxta* distribution (Figures 1D and 1D′). Conversely, the posterior neural tube population has a tight distribution of nuclear *tbxta* with a mean normalized intensity close to 0 and a broader nuclear *sox2* distribution (Figures 1E and 1E′). On the other hand, the NMps are distinct from both populations as they have broad marginal distributions of both nuclear *sox2* and *tbxta* (Figures 1F–1F″).

Next, we quantified the nuclear *sox2* and *tbxta* levels within the NMp population across the different somitogenesis stages. In the gene expression scatterplots (Figure 1J), we find that most NMps are *sox2*+_low_*tbxta* + _low_. However, at 24ss, we also find a greater number of *sox2*+_int_*tbxta* + _int_ NMps (Figure 1J 24ss red box), reflecting a transient increase in the transcriptional heterogeneity of the NM gene expression states. We then quantified the number and position of NMps at each stage across multiple individual tailbud samples. We found significant variation in the position (Figures 1G–1I) and number (Figure 1K) of NMps across samples at all stages under study. Notably, peak variability in NMp number occurred at the 24ss (Figure 1K). Taken together, our analysis demonstrates that a transient phase of increased heterogeneity in *sox2* and *tbxta* expression states occurs around 24ss. This closely matches the developmental stage at which labeled NMps contribute to both spinal cord and paraxial mesoderm (Attardi et al., 2018) and therefore suggests that the increased heterogeneity precedes the commitment to either NM fate.

### Analysis of 18ss scRNA-seq data reveals a peak in the critical index and transcriptional noise index in the NMp population relative to its derivatives

A second prediction of the transition state model is that cells should explore a larger region of gene expression space prior to cell fate commitment as the progenitor basin flattens, resulting in a more dispersed ‘cloud’ of points in state space (Huang, 2009). Consequently, cell population heterogeneity would be expected to increase, with between-gene variation decreasing as cells up-regulate groups of either neural or mesodermal genes in coordinated fashion (Mojtahedi et al., 2016). To assess this in the context of zebrafish NMps *in vivo*, we made use of a published single-cell RNAseq dataset at 18ss (Wagner et al., 2018).

**Figure 2:**
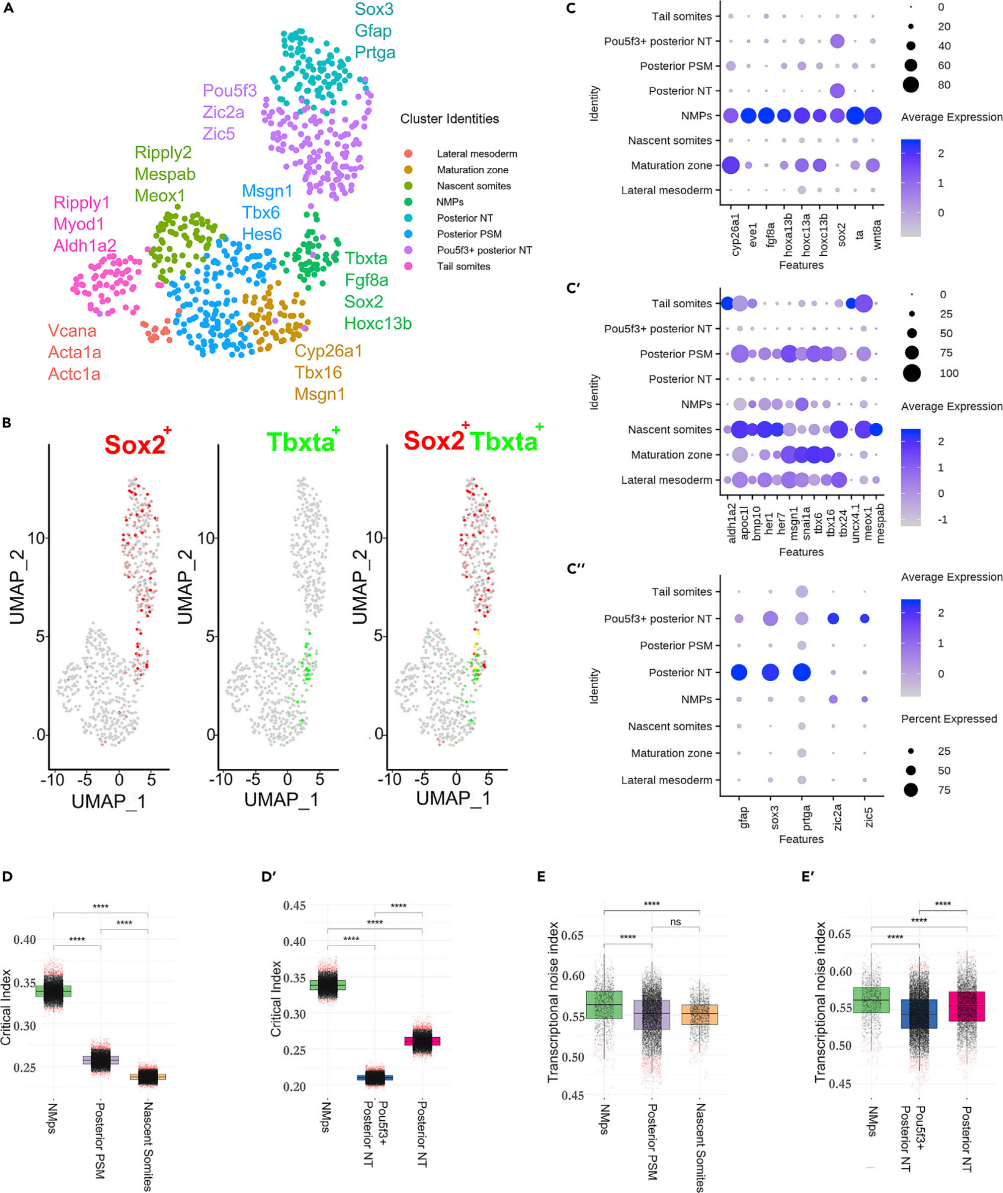
Analysis of 18ss scRNA-seq data reveals a peak in the critical index and transcriptional noise index in the NMp population relative to its derivatives (A) UMAP embedding showing the 8 tailbud clusters at 18ss alongside the key differentially expressed genes used for manual annotation. UMAP: uniform manifold approximation and projection. (B) UMAP embedding in (A) colored by *sox2* and *tbxta* expression and *sox2*-*tbxta* together to illustrate co-expression (in yellow). (C–C″) Dot plots displaying the expression of differentially expressed genes from the tailbud (C) NMp cluster, (C′) Mesodermal clusters and (C″) Neural clusters. (D–D′) Distribution of the critical transition index I_c_ calculated using marker genes of each cluster along the mesodermal (D) and neural (D′) branches. A bootstrapping procedure was applied in calculating I_c_ to account for the differences in cell number between cell clusters. Wilcoxon-Mann-Whitney unpaired two-sample test ∗∗∗∗p value < 0.0001; ns, not significant. (E–E′) Distribution of pairwise cell-to-cell distances/transcriptional noise along the mesodermal (E) and neural (E′) branches. Wilcoxon-Mann-Whitney unpaired two-sample test ∗∗∗∗p value < 0.0001; ns, not significant.

First, we reanalyzed the scRNA-seq data using an independent dimensional reduction and clustering approach to obtain the 8 tailbud subclusters that include the NMps and their derivatives (STAR Methods). We evaluated the robustness of our clustering approach which aimed at minimizing the clustering uncertainty arising from the small number of identified cells in the dataset (6959) (STAR Methods; Figures S3 and S4). We include the expression of selected differentially expressed genes for the neural, mesodermal and NMp clusters as dot plots (Figures 2C–2C″) and provide information on marker gene expression for all 8 clusters in Table S3. In support of our manual annotation of the NMp cluster, we find that most of the *sox2*+*tbxta*+ co-expressing cells are found within the NMp cluster, and the NMp cluster is sandwiched between two neural clusters and five mesodermal clusters (Figures 2A and 2B). This is consistent with *sox2* and *tbxta* emerging as differentially expressed genes in this cluster (Table S3). In addition, we validated a subset of the identified NMp marker genes experimentally via HCR and find that they are all expressed within the NMps within the tailbud (Figure S6), supporting the robustness of our *in silico* analysis.

We find that the NMp cluster is enriched for the posterior Hox genes *hoxc13b*, *hoxc13a* and *hoxa13b*, with avg_log2FC of 1.02, 0.94 and 0.78 respectively (Table S3). Avg_log2FC measures the log fold-change of the average expression of these genes in the NMp cluster versus the other clusters (Stuart et al., 2019). In terms of signaling pathways, *wnt8a* and *fgf8a* appear as the top two genes enriched in the NMp cluster, both of which are actively involved in NMp maintenance and differentiation (Goto et al., 2017; Row et al., 2016). Notably, *fgf8a* is expressed in >80% of cells in the NMp cluster (PCT1 = 0.816) and <8% of cells in all other clusters (PCT2 = 0.075). Finally, our analysis also identified *cyp26a1*, a retinoic-acid degrading enzyme that safeguards the Wnt/*tbxta* positive feedback loop, with an avg_log2FC of 1.02 (Martin and Kimelman, 2010).

Besides identifying the known molecular players in NMp differentiation, we also uncovered numerous other genes involved in a diverse range of processes. Genes with annotations that implicate their roles in signaling pathways feature prominently and include *wls*, *wnt8-2*, and *depdc7* for Wnt, *angptl2b* and *her12* for Notch, *nog2* and *id3* for BMP and *fgf4* for FGF signaling. Also, three genes were annotated with cytoskeleton-associated processes (*tagln3b* and *enc1* have actin-binding activity and *kif26ab* regulates microtubule motor activity), two possess histone deacetylase binding activity (*znf703* and *kdm6a*) and another two are associated with ubiquitination (*traf4a* and *ubl3a*). Interestingly, *foxd3*, a neural crest marker, emerged as a candidate that is enriched in the NMp cluster, with an adjusted pvalue of 1.92 × 10^−6^. It is expressed in >20% of cells in the NMp cluster and <4% of cells across all the other 7 clusters (Table S3). This observation confirms the results of recent study that revealed a common transcriptomic signature of the neural crest and NM populations (Lukoseviciute et al., 2021).

Cell fate decision making has been proposed to be a critical transition event, with both these features captured in a single Critical Transition Index that has previously been shown to predict a cell fate decision making event within blood progenitors as they commit to either myeloid or erythroid lineages (Mojtahedi et al., 2016). In similar vein, we computed the critical indices for the NMp, neural (pou5f3+ posterior NT and posterior NT) and mesodermal (posterior PSM and tail somites) clusters (Figures 2D and 2D′). Along both the neural and mesodermal differentiation trajectories, the NMp cluster cells have the highest critical index, which is consistent with a cell population undergoing a dynamical bifurcation.

Next, we assessed the level of transcriptional noise in the population, which measures the pairwise cell-cell distances (Mohammed et al., 2017). We observed that the NMp cluster cells have a higher transcriptional noise relative to the other cell populations (Figures 2E and 2E′). Therefore, both quantitative indices indicate that the NMp cell population is noisier than either the neural or mesodermal progenitor states that derive from the NMP population, lending support the hypothesis that NMps are approaching a critical transition at 18ss.

### Gene expression imputation and the construction of a composite map via ZebReg demonstrates a peak in the NM index entropy at 24ss

**Figure 3:**
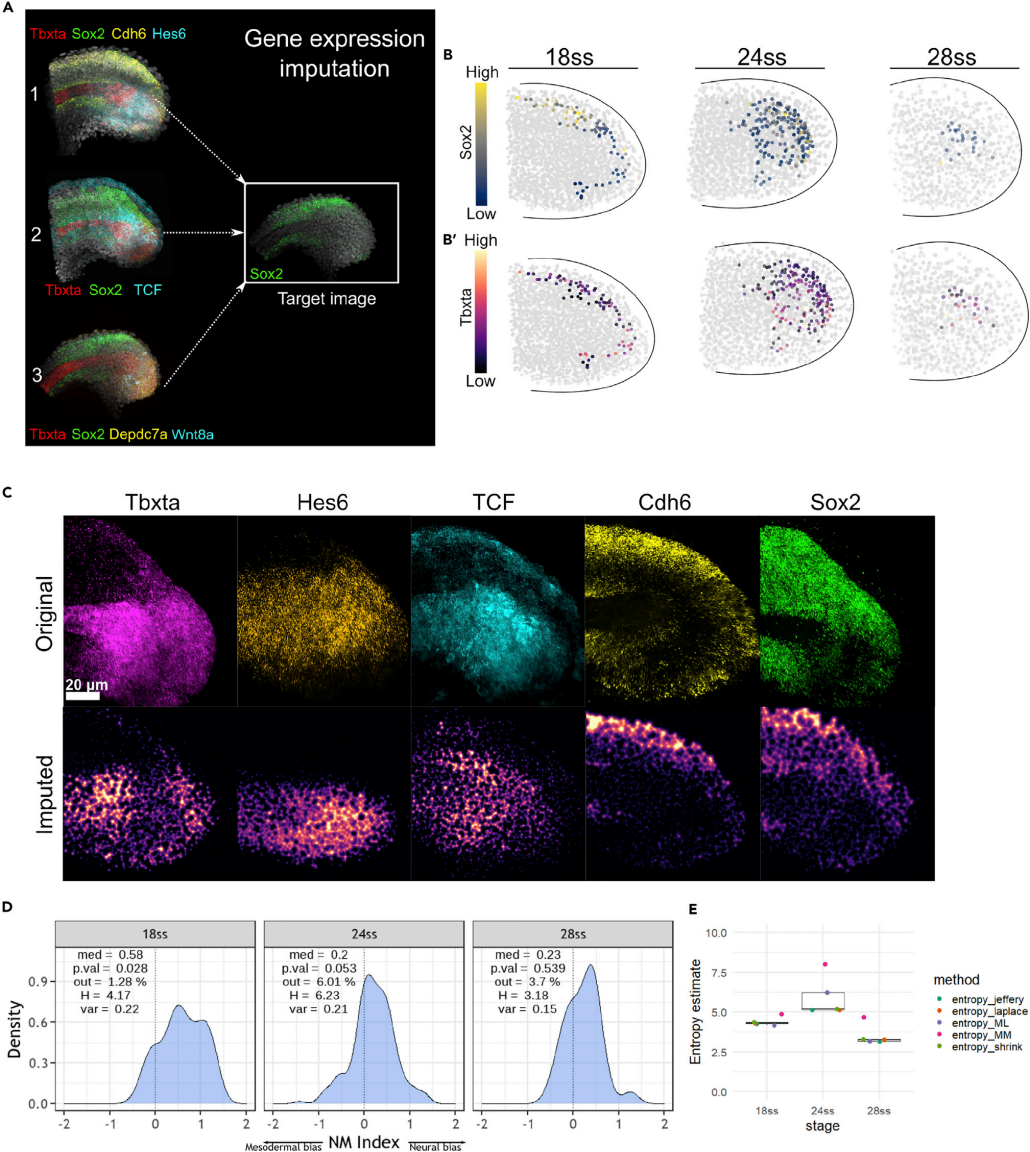
Gene expression imputation and the construction of a composite map via ZebReg demonstrates a peak in the NM index entropy at 24ss (A) Application of ZebReg for the imputation of multiple genes onto a target composite image. In the panel, *tbxta*, *cdh6*, *hes6*, *tcf*, *depdc7a* and *wnt8a* are imputed onto a target image that is stained only for *sox2*. *sox2* is the common color channel used to assist the alignment of the source images onto the target image. In this example, the resultant target image has 7 distinct color channels. (B–B′) Coloring *in silico* NMps in the 18ss, 24 and 28ss composite maps by (B) *sox2* expression (B′) *tbxta* expression levels. (C) The top ‘Original’ row depicts the 2D projections of the HCR data at 18ss. The bottom ‘Imputed’ row depicts the corresponding expression of these genes in the target composite image. (D) NM index density distributions computed from the 8-gene composite maps at 18ss, 24 and 28ss. Negative values of the NM index indicate mesodermal bias, whereas positive values indicate neural bias. med: median; p.val: pvalue for the Shapiro-Wilk test; out: outlier percentage; H: empirical entropy estimate; var: variance. (E) Entropy estimates of the NMp index, with the estimation of the SE obtained via jackknife resampling. The entropy estimates consistently peak at 24ss. entropy_jeffrey: Dirichlet-multinomial pseudocount entropy estimator (Dirichlet) with Jeffrey’s prior; entropy_laplace: Dirichlet with Laplace’s prior; entropy_ML: empirical maximum likelihood estimator; entropy_MM: Miller-Madow estimator; entropy_shrink: James-Steintype shrinkage estimator.

Our observation that the number and position of NMps vary extensively between stage-matched embryos, especially at the 24ss, suggests that there is significant variability in *sox2* and *tbxta* expression within the NMps. Consequently, fixed measurements of gene expression from a single sample alone would be inaccurate as it can only give an instantaneous snapshot capturing one out of many different gene expression states that the NMp population can potentially explore. To leverage the gene expression information across multiple tailbud samples, we developed a tool called ZebReg that takes images of stage-matched zebrafish tailbud samples as inputs, converts them into point clouds and registers the point clouds together to construct composite gene expression target maps (Figure 3A).

We first used ZebReg to impute the expression of 8 genes into three composite maps, one for each stage at 18ss, 24 and 28ss. Each composite map was constructed by combining the gene expression of 6 different images across different HCR experiments (Figure S8 and Table S2), and each map displays the expression of *sox2* (Figure 3B), *tbxta* (Figure 3B’) and 6 other neural or mesodermal marker genes selected from our scRNA-seq analysis within a target point cloud. As these maps contain spatial information of the tailbud cells, we could identify the *in silico* NMps by virtue of their *sox2* and *tbxta* co-expression as well as their locations on the composite map (Figure S12). These *in silico* NMps in the composite maps were also found to be within the NMp regions in our probability map which were identified via a different approach (Figure S14).

To assess the fidelity of our gene expression imputation procedure, we performed a gene-by-gene qualitative inspection of the spatial expression patterns in the composite maps to the corresponding patterns observed in the original HCR images at 18ss. A visual comparison between the imputed and original images demonstrates a strong resemblance in their expression patterns (Figure 3C). For instance, *sox2* and *cdh6* are expressed strongly in the posterior neural tube and hypochord but not in the notochord. *tbxta* is expressed strongly in the notochord progenitor zone and the dorsal PW. Thus, the overall visual correspondence between the original and imputed gene expression images is evidence that ZebReg has aligned these images appropriately, at least when assessed on a qualitative level. We also performed additional *in silico* validation experiments and demonstrated that ZebReg also preserves the quantitative relationships between genes (STAR Methods).

In a previous study involving the generation of NMps from mouse embryonic stem cells *in vitro* (Edri et al., 2019), the authors developed an NMp index that measures the neural and mesodermal potentials of different cultured cell populations. In similar vein, we used the composite maps to construct an ‘NM index’ which combines the information across 8 genes (STAR methods) and differs from the NMp index by Edri et al. in several important dimensions (Table 1). We plotted the NM index distributions for the NMps in the three composite maps (Figure 3D). These distributions reflect the neural/mesodermal biases of the *in silico* NMps in these three stages, and cells can be classified as being either neural-biased, mesoderm-biased or indecisive based on their NM index value (Figure S13A). We find that there is a consistent neural bias in the NMps across all three stages which is reflected by the median NM index value.

**Table 1:** Differences in the construction of the NMp indices

Comparison	NMp index (Edri et al., 2019)	NM index
How is the index defined?	Relationship between the neural averaged value/potential and the mesodermal averaged value/potential. This construction is similar to the analysis of the Neural and Mesodermal indices (Figure S13C)	A summary statistic defined as the difference between the neural and mesodermal indices.
How are the gene expression values normalised?	*Z* score normalization	Min-max normalization
How many genes (including Sox2 and Tbxta) are used in the construction of the index?	19	8
Are genes categorised?	Yes. There are 4 neural genes and 15 mesodermal genes. The effect of categorization is that neural genes contribute only to the neural average value, and mesodermal genes contribute only to the mesodermal average value.	No. All 6 genes can contribute to both indices (sox2 and tbxta contribute only to the Neural and Mesodermal indices respectively).
Do genes contribute equally to the index?	Yes. For instance, the neural potential is an average of the 4 neural genes (including sox2)	No. Sox2 and tbxta has the highest contribution to the index, with the other 6 genes weighted for their correlation to each gene. The stage-wise correlations were obtained from our segmented NMp HCR data (Figure S10B)
How were genes selected?	Supervised selection by experts.	Unsupervised approach based on analysis of scRNA-seq data.
Is the index defined at a single-cell level?	No. The index is defined for entire cellular populations. mRNA from bulk samples were extracted and quantified via qRT-PCR.	Yes. Each cell is assigned a value of the index based on imputed gene levels.

To quantify the NM heterogeneity of the *in silico* NMps between these stages, we computed a series of Shannon entropy estimators. Examining the empirical maximum-likelihood entropy estimator (H) with the natural unit of information (nat), we observed a surge in value at 24ss with H = 6.23 nat, compared to the neighboring values of H = 4.17 nat at 18ss and H = 3.18 nat at 28ss (Figure 3E). This increase in entropy followed by a decline was also observed in the other entropy estimates (Figure 3E). Thus, our data suggest that the NMp population heterogeneity, measured by the intercellular entropy, peaks at 24ss.

### ZebReg’s composite maps reveal that the number of rebellious cells peak at 24ss

We further compared the canonical Wnt signaling activities and eventual cell fates (neural or mesodermal) of the *in silico* NMps against their NM gene expression states (NM index levels). To monitor the downstream transcriptional activity of canonical Wnt signaling, we probed GFP RNA levels produced from a transgenic line that expresses GFP downstream of seven multimerized TCF/LEF binding sites (Moro et al., 2012). Given that our data consist of fixed snapshot images of the NMps, we cannot follow the differentiation of single NMps over time and thus, do not have direct information of their prospective fates. Nevertheless, snapshot images can inform us of NMp fates due to their specific developmental features. In a previous study by Attardi et al., photolabeling of the zebrafish NMps followed by single-cell lineage tracing in a light-sheet dataset demonstrated that these are mono-fated and spatially segregated progenitors (Attardi et al., 2018). Consequently, a reliable fate map of the tailbud NMps can be constructed, where the fate of an NMp can be inferred from its spatial location at the mid to late somitogenesis stages. In addition, as NMps have low levels of proliferation (Attardi et al., 2018, Figures S11A, S11B, S11D, and S11E) and apoptosis throughout 18ss–30ss (Figures S11A and S11C), we can be confident that we are following the differentiation trajectory of an identified NMp over time.

**Figure 4:**
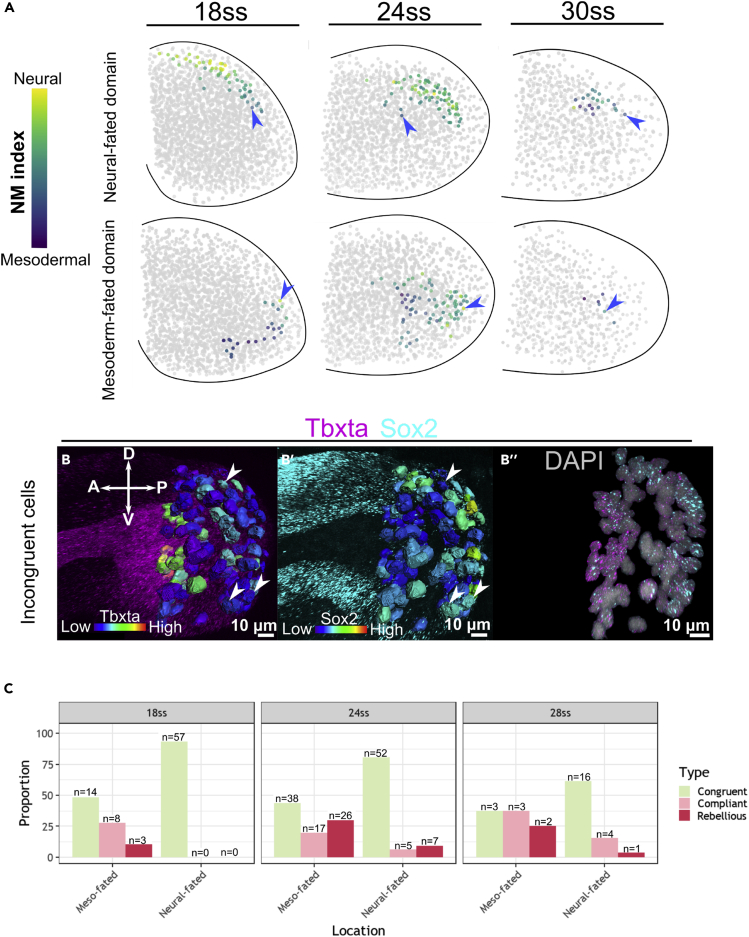
ZebReg’s composite maps reveal that the number of Rebellious cells peak at 24ss (A) Demarcation of the neural-fated and mesoderm-fated domains in the composite maps. Non-NMps are colored gray, whereas NMps are colored according to their NM index levels. Blue arrows mark incongruent cells. (B–B″) HCR stains of a representative zebrafish tailbud at 24ss for *tbxta* and *sox2*. Segmented surfaces correspond to NMps which are colored by the expression levels of *tbxta* (B) and *sox2* (B′). Nuclear signals for *sox2* and *tbxta* are shown to illustrate co-expression (B″). Arrow heads mark Incongruent cells. (C) Proportion of Congruent, Compliant and Rebellious cells in the mesoderm-fated and neural-fated domains at 18ss, 24 and 28ss. At each stage, summing up the number of Compliant and Rebellious cells yields the number of Incongruent cells.

Given the critical relationship between an NMps’ spatial location and its cell fate, we defined approximate neural-fated and mesodermal-fated domains in our ZebReg composite maps following the fate map of Attardi et al. and assessed the NM index levels of the NMps within these domains (Figure 4A). We found cells with NM gene expression profiles that are inconsistent with their prospective fates and labeled these cells as ‘Incongruent’. Conversely, cells with compatible state-fate relationships are labeled ‘Congruent’. We observed Incongruent cells with low/high NM index levels residing in the neural/mesoderm-fated domains (Figure 4A blue arrows). As *sox2* and *tbxta* are the primary orchestrators of the neural and mesodermal gene expression programs respectively, these ‘incongruent’ cells are also found in our original HCR images as *sox2* (*tbxta*)-high NMps within the neural (mesoderm)-fated domains (Figure 4B).

Incongruent cells can be further classified as ‘Compliant’ or ‘Rebellious’ depending on whether their Wnt signaling activities (*tcf* expression levels) are consistent or inconsistent with their NM gene expression states (Table S1). We quantified the proportion of Compliant, Rebellious and Congruent cells in the mesodermal and neural-fated domains of our three composite maps (Figure 4C). At 18 and 28ss, with the exception of the 28ss mesoderm-fated domain, most cells are Congruent. Also, more Incongruent cells are found in the mesoderm-fated domain than the neural-fated domain. However, at the 24ss, we find a greater number of Incongruent cells (Compliant and Rebellious) than Congruent cells in the mesoderm-fated domain. Specifically, the number of Rebellious cells in the mesoderm-fated domain peaks at this stage. Thus, consistent with the transition state model, we find a loosening of the relationship between cell state and fate as reflected by the increase in the number of rebellious NMps at the 24ssprior to their commitment to the NM fate.

## Discussion

Zebrafish tailbud NMps have proven to be an attractive *in vivo* system to assess the transition state hypothesis. Specifically, we investigated whether a transient window of elevated stochasticity in gene expression precedes the NMp differentiation event at around 24ss. Our study supports the existence of an *in vivo* transition state via 3 main lines of evidence. First, in our single-nuclei *in situ* HCR stains of Sox2 and Tbxta, we found an increased variability in NMp cell number (Figure 1K) as well as gene expression heterogeneity at 24ss (Figure 1J). Second, analysis of the high-dimensional scRNAseq dataset at 18ss showed that the critical transition index and transcriptional noise index peak in the NMps (Figures 2D and 2E), indicating that it is a population with elevated noise levels. Third, by integrating multiple HCR stains into composite maps with the ZebReg image registration tool and then computing the NM index distributions, we found an increase in intercellular entropy in the NMp population specifically at 24ss (Figure 3E) and also document the existence of Rebellious cells *in vivo* (Figures 4B and 4C).

Our discovery of rebellious cells in the ZebReg composite maps recapitulates the finding by Mojtahedi et al. in their *in vitro* study on the differentiation of a multipotent hematopoietic cell line (Mojtahedi et al., 2016). Rebellious cells emerge at day 3 post-treatment as cells that express an erythroid profile when stimulated with Granulocyte macrophage colony stimulating factor/IL-3 or a myeloid profile when stimulated with erythropoietin. Eventually, cells disappear at day 6 post-treatment. In addition, ‘edge’ cells were identified in cancer cell lines as cells that adopt a gene expression profile that is different from the average profile in the population distribution (Li et al., 2016). This phenomenon is not unprecedented *in vivo*. In an earlier study on Xenopus embryonic development (Wardle and Smith, 2004), cells that express a lineage marker at the ‘wrong’ place, such as Goosecoid expressing cells in the ventral instead of the dorsal region of the embryo, were labeled‘rogue’ cells to indicate their abnormal expression profile. These cells appear more frequently in the early gastrula stage and reduce in frequency at the late gastrula stage. In both cases, these rebellious/rogue cells are proposed to ‘fit in or die trying’ - they would either die by apoptosis or transdifferentiate to adopt the appropriate gene expression profile if rescued by delivery of the appropriate signal or through interactions with neighboring cells via the community effect.

One of the key results is the transient increase in NMp cell number and gene expression variability at 24ss (Figures 1J and 1K). What could be the plausible explanation for this phenomenon? The spike in the number of NMps is unlikely to be the result of cellular proliferation occurring around 24ssdue to very low proliferation in the NMP region throughout the differentiation process (Bouldin et al., 2014; Zhang et al., 2008; Figure S11). We also assessed for the possibility of temporal differences in apoptotic levels throughout tailbud development and found consistently low levels of apoptosis as well (Figure S11). Instead, we propose that this arises because NMps are undergoing a critical transition around this developmental window. During NMp differentiation, stochastically fluctuating levels of *Sox2* and *Tbxta* could result in the broad range of distributions observed within the NMp population, leading to the identification of fewer or more sox2+tbxta+ cells in our fixed imaging analysis. More Sox2+tbxta+ cells could be identified than before as Sox2-Tbxta+ cells could upregulate Sox2 to reach detectable levels of expression (or vice versa). Thus, the peak in cell number variability and gene expression heterogeneity at 24ss are interrelated, coinciding with the NMps’ entry into the transition state as cells sample a broader set of Sox2 and Tbxta states. This is consistent with the emergence of sox2+_int_ tbxta + _int_ cells at 24ss (Figure 1J, red box 24ss). The strongest support for this explanation comes from previous lineage tracing studies demonstrating that NMps begin to contribute to the spinal cord only after the 24 somite stage, as labeled descendants of the NMP region only contribute to post-24 somite regions of the tail (Attardi et al., 2018). As the critical transition theory posits a decrease in correlation of cellular gene expression states between cells prior to their differentiation (Mojtahedi et al., 2016), this observation extends our knowledge of the mechanisms underpinning the biological timing of NMP differentiation *in vivo*.

To construct the gene expression composite maps, we developed an image registration tool, ZebReg, which employs a point-based registration approach by converting the centroids of segmented zebrafish nuclei surfaces into points. Image registration tools built for the study of zebrafish embryonic development are rare. A gene expression atlas was constructed for the early embryonic shield stage (Castro-González et al., 2014) and another registers reporter gene activities of embryos at prim-20 and long-pec stages (Gehrig et al., 2009). To the best of our knowledge, there is no existing image registration tool built for the purpose of aligning zebrafish embryos at segmentation stages (10.33 hpf to 24 hpf) and for the quantification of gene expression intensities in the zebrafish tailbud. We adopted a rigid transformation approach for ZebReg as th e developing notochord is a prominent morphological landmark in the zebrafish tail, which allowed us to crop the volumetric images easily to maintain a consistent field of view and thus simplify the registration task considerably (Hajnal et al., 2001). Also, zebrafish tailbuds of around the same stage are reasonably consistent in their morphology and there were no obvious shrinkage artifacts arising from the experimental procedure. In cases where significant biological and technical variation exist (Fowlkes et al., 2008), a pipeline that uses non-rigid transformation methods for correction of these distortions would be required (Keszei et al., 2017).

The development of the NMp index was critical for the quantitative analysis of the composite maps. One feature that was highlighted from the index is the neural bias in the NMps across all three developmental timepoints. Given that the NM index is constructed using correlations, it may give a skewed estimate if the NMp genes chosen from the scRNA-seq dataset for HCR validation are all highly correlated toward either the neural (*Sox2*) or mesodermal (*Tbxta*) fates. To assess this possibility, we re-examined the correlation data of all 6 genes with *Sox2* and *Tbxta* (Figures S13E and S13E′). We found that for both genes at all three stages, the correlation values spanned a range of positive and negative values, although there is a slight bias toward a positive correlation toward Sox2 and negative correlation toward*Tbxta* at the later stages. Thus, the reported neural bias is not a consequence of examining genes specifically correlated positively to *Sox2* or negatively to *Tbxta* only. In addition, our primary observation of a transient peak in entropy at 24ss remains unchanged when we computed the naive index (Sox2-Tbxta). Indeed, the correlation between the naive index and the NM index is strong (around 0.8–0.9 for all 3 stages). In fact, when substituting Tbxta for TCF and Cdh6 for Sox2, given the relatively strong correlation of each member of the pair with each other, the conclusion remains unchanged. Another approach taken was to compute the NM index in a similar fashion to Edri et al. (2019) by categorising genes into either neural or mesodermal categories. When the NM index was computed as (Sox2 + Cdh6) - (Tbxta + TCF), the peak in entropy at 24ss is in fact even higher than the corresponding entropy value of the naive index at 24ss. Therefore, we can identify the peak in cell-cell variability at 24ss, where variability here is quantified in terms of the entropy of the NM index distribution, in a manner that is robust to the precise choice of genes for the NM index. We believe that this robustness is due to the increased gene-gene correlation within the individual neural and mesodermal modules, as well as greater antagonism between both modules as NMps undergo a critical transition around 24ss.

In the construction of the composite maps at 18ss, 24 and 28ss, the mesodermal and neural-fated domains comprise varying numbers of cells, which is a consequence of the developmental dynamics of the zebrafish NMps (Figure 1K). Our finding that the proportion of rebellious cells (Figure 4C) is highest at 24ss within the mesoderm-fated domain extends a recent study on the connection between morphogenetic movements and mesoderm fate acquisition in the zebrafish NMps (Kinney et al., 2020). *Sox2* and canonical Wnt co-expression in mesoderm-fated NMps primes these cells toward both neural and mesodermal fates and acts as a developmental checkpoint that traps these cells in a poised, intermediate state. This intermediate state where EMT is delayed resembles a hybrid EMT transition state found in cells with high potential for metastasis (Yang et al., 2020). In fact, *tbxta* (Brachyury) is a driver of EMT in various tumors and is correlated with metastatic activity and the acquisition of a mesenchymal phenotype (Chen et al., 2020). Thus, our work emphasises a strong connection between the hybrid EMT transition state expressing multiple intermediate cell states (Sha et al., 2019) and the neural-mesodermal transition state (Steventon and Martinez-Arias, 2017). The correspondence between morphological fluctuations and the entry into a transitory state was also recently proposed in a study on hematopoietic stem and progenitor cells (Moussy et al., 2017). As NMps exit the transition state and differentiate into the neural or mesodermal fates, the heterogeneity in *sox2* and *tbxta* expression is resolved as NMps adopt either a high *sox2*/low *tbxta* (neural) or high *tbxta*/low *sox2* (mesodermal) expression profile (Figures 1D–1F). This is consistent with the proposed role of Sox2 and Bra protein level ratios dictating the specific cell movements associated with each lineage (Romanos et al., 2021).

Recent work on multipotent zebrafish neural crest cells suggests that at least a portion of the neural biased trunk neural crest (NC) progenitors arise from early neural biased zebrafish NMps at 5-6ss (Lukoseviciute et al., 2021). A similar conclusion was reached in multiple studies of *in vitro* human pluripotent stem cell-derived axial progenitors, demonstrating that the generation of trunk NCs involves an obligatory NMp intermediate (Frith et al., 2018; Hackland et al., 2019). We identified *sp5l*, *cdh6*, *znf703* and *foxd3* as differentially expressed genes of the NMp cluster; all of which have important roles in neural crest specification. In addition, many differentially expressed genes identified from the NMp cluster are involved in signaling pathways (FGF, Wnt, BMP) and the synergistic action of these pathways play a critical role in neural crest differentiation (Sauka-Spengler and Bronner-Fraser, 2008). When we photolabelled the dorsal PW (NMp region) at 18ss and tracked these cells until 28ss, we noticed that the anterior photolabels in the dorsal neural tube appear to be emigrating away whilst the posterior labels do not show signs of migration (Figure S11D). 24 h later, the photolabels were found to have spread more anteriorly, with the anterior labels appearing more dispersed ventrally. When we photolabelled the dorsal PW at 28ss, we also found similar photolabels in the dorsal neural tube 24 h post-photolabeling (Figure S11E). The localization of the labels in the dorsal neural tube alongside the anterior pattern of cell migration strongly suggest that the differentiation of the NMp-derived neural progenitors into the trunk NC progenitors continues throughout somitogenesis and occurs even as we approach the end of somitogenesis. Therefore, we extend the observation made by Lukoseviciute et al., providing support for an NMp to trunk NC progenitor lineage that occurs even in the later tailbud NMp population.

To the best of our knowledge, our work is the first to directly catalog the transient surge in heterogeneity in mRNA expression *in vivo* during an endogenous differentiation event in a wildtype vertebrate species. Whilst several studies have proposed mechanistic models to explain the relationship between transcriptional heterogeneity and cell fate commitment (Antolović et al., 2017; Pina et al., 2012) and even functional pluripotency (MacArthur and Lemischka, 2013), our study was not designed to discriminate between these causal models. Instead, we focused on assessing the association between cell fate commitment and the increase in gene expression heterogeneity *in vivo*. Future work, outside the scope of this article, is necessary to fill in the mechanistic details that generate these heterogeneities during cell fate transitions *in vivo*.

Taken together, our work supports the existence of a transition state within an endogenous cell fate decision making event. Recognising the functional importance of transcriptional stochasticity and non-genetic heterogeneities during differentiation has important practical consequences. It drove the discovery that regulators of transcriptional noise may play a general role in the acquisition of malignancy by modulating the balance between proliferation and differentiation (Domingues et al., 2020), and may be an important dimension to consider when improving the efficacy of mesenchymal stem cell-based therapies (McLeod and Mauck, 2017; Pacini, 2014). Seen alongside the evidence presented from other systems, it becomes increasingly plausible that the transition state is not an idiosyncrasy of *in vitro* culture conditions or a peculiarity of cancer models. We await future developments on whether the critical behaviors predicted in the transition state model are a universal characteristic of cell state transitions in biological systems *in vivo*.

### Limitations of the study

The colored ICP (cICP) algorithm employed in ZebReg will not be able to align point clouds exactly, as zebrafish tailbuds will inevitably differ from one another in their nuclei position and gene expression intensities. Instead, for each nucleus from the source image, ZebReg can, at best, map it to its most similar cell counterpart in the target image, based on their proximity to each other and similarity in expression of a reference gene. For multiply mapped and unmapped target points, ZebReg imputes their gene expression intensities by taking the average intensities of each point’s k-nearest neighbors (k = 5). This approach assumes a degree of spatial autocorrelation in gene expression intensities of a point with its neighbors. During the transition state where cell-cell correlation decreases, our approach may underestimate the extent of cellular heterogeneity in the population due to the application of an averaging procedure.

In our work, we adopted a descriptive, fixed imaging-based approach toward interrogating the level of gene expression heterogeneities during NMp differentiation. Whilst the peculiarities of the zebrafish NMp model enabled us to infer cellular fates from cellular positions, without adopting a live imaging approach, we were unable to document the details of the transcriptional dynamics around 24ss (Weinreb et al., 2018). Zebrafish embryos have been amenable to live RNA imaging using various techniques such as the MS2 labeling system (Campbell et al., 2015), 3′ poly(A) tail labeling system (Westerich et al., 2020) and molecular beacon sensors (Li et al., 2017) due to its optical transparency. Thus, future work could perform live imaging of *sox2* and *tbxta* mRNAs to monitor the changes in transcription dynamics around 24ss as NMps enter and exit the transition state.

## STAR★Methods

### Key resources table

**Table:** 

REAGENT or RESOURCE	SOURCE	IDENTIFIER
**Antibodies**		

Mouse monoclonal Anti-Histone H3 (phosphor S10)	Abcam	Cat #Ab14955; RRID:AB_443110
Rabbit polyclonal Anti-caspase3	Abcam	Cat #Ab13847; RRID:AB_443014
Secondary anti-mouse Alexa Fluor A488	ThermoFisher Scientific	Cat # A32723; RRID:AB_2633275
Secondary anti-rabbit Alexa Fluor A633	Invitrogen	Cat #A21071; RRID:AB_2535732

**Chemicals, peptides, and recombinant proteins**		

4% paraformaldehyde (PFA)	Sigma	CAS no: 30,525-89-4
Agarose, low gelling temperature	Sigma	A9414
Dulbecco’s Phosphate Buffered Saline (PBS)	Sigma	D8537-500ML
DAPI	Sigma	CAS no: 28718-90-3
EDTA	Sigma	CAS no: 60-00-4
Triton-X	Sigma	CAS no:9002-93
Fetal bovine serum (heat-inactivated)	ThermoFisher Scientific	10437028
Bovine serum Albumin	Sigma	A7906-10G
Methylcellulose	Sigma	M0512
RNaseA	QIAGEN	19101
SSC buffer	Scientific Laboratory Supplies	S6639-1L
Methanol	ThermoFisher Scientific	CAS no: 67-56-1
VECTASHIELD Antifade mounting medium	Vector Laboratories	H-1000-10
Tween-20	ThermoFisher Scientific	AAJ20605A

**Critical commercial assays**		

*In situ* HCR v3.0	Molecular Instruments	N/A
QIAquick PCR purification kit	Qiagen	28104
SP6 mMessage mMachine kit	Invitrogen	AM1340

**Deposited data**		

RNA seq	NCBI Gene Expression Omnibus	GEO: GSM3067194
ZebReg Code	This paper	https://doi.org/10.5281/zenodo.7053174

**Experimental models: Organisms/strains**		

Wildtype Zebrafish embryos - Tüpfel long fin (TL)	European Zebrafish Resource Center	ZDB-GENO-990623-2
Wildtype Zebrafish embryos - AB	European Zebrafish Resource Center	ZDB-GENO-960809-7
Transgenic zebrafish embryos - Tg(7x*TCF*- Xla.Sia:GFP)	Steven Wilson Lab; Moro et al., 2012	ZDB-TGCONSTRCT-110113-1

**Oligonucleotides**		

Tbxta HCR probes	This paper	See Table S4
Sox2 HCR probes	This paper	See Table S4

**Recombinant DNA**		

Hsp70L:p2a-NLS kikGR	Benjamin Martin Lab; Row et al., 2016	ZDB-TGCONSTRCT-160321-3

**Software and algorithms**		

Imaris (v9.2.1)	Bitplane	https://imaris.oxinst.com/
Python (v3.8)	Python Software Foundation	https://www.python.org/
Open3D (v0.11.0)	Zhou et al., 2018	http://www.open3d.org/

**Other**		

NanoDrop 2000c Spectrophotometer	Thermo Scientific	13400411
35 mm glass bottom dish	MatTek	P35G-1.5-10-C
Inverted confocal Microscope	Leica	SP8
Inverted confocal Microscope	Zeiss	LSM700
KpnI-HF enzyme	NEB	R3142L

### Resource availability

#### Lead contact

Further information and requests for resources should be directed to and will be fulfilled by the lead contact, Ben Steventon (bjs57@cam.ac.uk).

#### Materials availability

HCR probe sequences for *sox2* and *tbxta* are documented in the supplementary file.

### Experimental model and subject details

#### Zebrafish husbandry

All zebrafish procedures were conducted under the Animals (Scientific Procedures) Act 1986Amendment Regulations 2012, following ethical review by the University of Cambridge Animal Welfare and Ethical Review Body (AWERB). Wildtype lines used are either Tüpfel long fin (TL), AB/TL or AB. The Tg(7x*TCF*- Xla.Sia:GFP) reporter line (Moro et al., 2012) was provided by the Steven Wilson laboratory. All embryos obtained were obtained and raised in standard E3 media at 28°C. Embryos were staged according to Kimmel et al. (1995).

### Method details

#### Version 3 hybridization chain reaction (V3 HCR)

Zebrafish embryos at the required stages were fixed in 4% PFA in DEPC-treated, calcium and magnesium-free PBS at 4°C overnight. Embryos were then stained with V3 HCR (Choi et al., 2018). All hairpins were purchased from Molecular Instruments. All probes were purchased from Molecular Instruments except for *sox2* and *tbxta* which were manually designed. After the staining procedure, samples were counterstained with DAPI at a dilution of 1:1000 in 5xSSCT for 2 hours at room temperature. The tailbud region was cut out with a forceps and eyelash tool, and then mounted on a 35 mm glass bottom dish (MatTek) with the VECTASHIELD Antifade mounting medium for confocal imaging.

#### Quantification of nuclear gene expression intensities

HCR images were processed in Imaris (Bitplane). Unless otherwise stated, all *sox2*+*tbxta*+ HCR images were analyzed for the number of NMps as described in Figure S1. Segmentation of the posterior neural tube and notochord nuclei were conducted with reference to their known anatomical locations.

To normalize the Sox2 and Tbxta gene expression intensities in the different populations identified across samples (posterior neural tube, posterior notochord, NMps), we performed the following steps. For each sample imaged, we recorded the highest and lowest signal intensities of Sox2 and Tbxta amongst all segmented nuclei of that sample. If the signal intensities were derived from inappropriately segmented nuclei, they were discarded and the next highest or lowest signal intensities were used instead to avoid outlier values. These threshold intensity values were then used for min-max normalization of the signal intensities.

#### Immunostaining

Zebrafish embryos at the required stages were fixed in 4% PFA in DEPC-treated, calcium and magnesium-free PBS at 4°C overnight. Embryos were then co-stained with a 1:500 dilution of mouse anti-PH3 antibody (Abcam, ab14955) and 1:500 dilution of rabbit anti-caspase3 antibody (Abcam, ab13847), as described in Sorrells et al. (2013). Secondary anti-mouse Alexa Fluor 488-conjugated antibody and anti-rabbit Alexa Fluor 647-conjugated antibody were both diluted in 1:500 PDT solution and incubated with the samples overnight at 4°C. DAPI was added at the final step with a 1:1000 dilution in PDT and incubated for 2 h at room temperature for nuclear detection. Images were quantified in the 3/4D Image Visualization and Analysis Software Imaris 9.2.1 (Bitplane). The percentages of mitotic or apoptotic cells for each sample were calculated as the fraction of PH3+ or caspase3+ nuclei over the total number of nuclei in the tailbud, multiplied by 100.

#### Photolabeling with nuclear-targeted kikume

The hsp70L:p2a-NLS kikGR vector (Bouldin et al., 2015) was extracted from an overnight grown bacterial culture. Briefly, bacterial cells were collected via centrifugation and washed sequentially with the following 3 buffers: P1 containing 50 mM Tris-Cl at pH 8.2, 10 mM EDTA at pH 8.0, RNase A (QIAGEN); P2 (filter-sterilized) containing 0.8% NaOH and 1% SDS; P3 containing 3M KOAc that is adjusted to pH 5.5 with glacial acetic acid. Plasmid DNA was precipitated with 70% isopropanol and washed with 70% ethanol before resuspension in nuclease-free water.

The vector was linearized by restriction digestion with the KpnI-HF enzyme (NEB), and subsequently purified using the QIAquick PCR purification kit (Qiagen). The purified, linearized plasmid was transcribed at the SP6 promoter with the SP6 mMessage mMachine kit (Invitrogen), and lithium chloride precipitation was carried out for mRNA recovery. Quantification of the transcribed kikGR mRNA was performed on the NanoDrop instrument (Thermo Fisher).

One-cell stage zebrafish embryos were injected with the NLS-kikGR mRNA and then embedded in low gelling point agarose (Sigma) at 1% w/v in E3 media at the bottom of a MatTek 35 mm glass bottom dish. Photoconversion and image acquisition was performed on a Zeiss LSM 700 confocal microscope. Efficient, irreversible photoconversion of NLS-KikGR in the zebrafish embryos at mid-somitogenesis stages was carried out by scanning the 405 nm laser at 15% laser power for approximately 30 seconds in a region of interest.

#### Confocal microscopy imaging

Samples were imaged on either a Zeiss LSM700 inverted confocal or a Leica TCS SP8 inverted confocal at 10X, 20X or 40X magnification.

#### Analysis of scRNA-seq data

##### Preprocessing *18hpf scRNA-seq dataset*

The wildtype 18hpf zebrafish scRNA-seq raw counts dataset and the associated clusterIDs were downloaded from GEO with the accession number GEO:GSM3067194 (Wagner et al., 2018). First, outlier cells with log-transformed library and feature sizes more than 3 median absolute deviations (MADs) from the respective median metric values were removed. Genes that were not expressed in the dataset were filtered out. At this quality control threshold, most genes and cells were retained for downstream analysis, resulting in a dataset with 30296 genes x 6954 cells (381 genes and 8 cells discarded). The data was then converted into a Seurat 3.0 object (Stuart et al., 2019) for subsequent analyses. Cell cycle scoring and regression were performed in Seurat 3.0 using a set of cell-cycle associated genes for zebrafish (Lush et al., 2019), with the S.Score and G2M.Score as inputs to the vars.to.regress argument in the *SCTransform* function. Data normalization, scaling and the identification of the top 3000 most variable genes were also carried out using the *SCTransform* wrapper.

##### Low dimensional embedding and Louvain clustering

The normalised and scaled data was projected into low dimensional subspace via principal components analysis (PCA) with default settings for the *RunPCA* function. (Figure 2B). Following this, the uniform manifold approximation and projection (UMAP) embedding was implemented via the *RunUMAP* function. To perform clustering, groups of similar cells on the UMAP embedding were identified by generating a shared nearest neighbor (SNN) graph of the dataset with the *FindNeighbors* function, and then clustered using the Louvain algorithm with the *FindClusters* function at various resolutions. Subclustering on the tailbud cells was performed in similar manner to the above clustering procedure, with a resolution of 1 set for the FindClusters function. To examine the clustering results, clustering trees were plotted with the *clustree* package whilst the adjusted rand index and clustering entropy were implemented in the *mclust* and *NMF* packages respectively.

##### Identification of differentially expressed genes

For each cluster, supervised annotation was carried out by examining the marker genes identified by a Model-based Analysis of Single-cell Transcriptomics (MAST) and a Wilcoxon Rank-Sum test. The tests were carried out using the *FindAllMarkers* function in Seurat that compares cells in each cluster against all other remaining clusters. The function is set to return only positive markers for each cluster (only.pos = TRUE). Differentially expressed genes with an adjusted p-value less than 0.05 were retained for analysis. They were then sorted in order of priority, based on the log fold-change of the average expression between the cluster under study and the remaining 7 tailbud subclusters (avg_log2FC).

##### Robustness analysis of tailbud clustering assignments

To assess the robustness of our selection of the zebrafish tailbud cells from the 18hpf dataset, we employed a different approach than Wagner et al. (Wagner et al., 2018) by embedding the 6,954 cells in the 18hpf dataset into a Uniform Manifold Approximation and Projection (UMAP) space and using the Louvain community detection algorithm to identify clusters (Figure S3A).

We first assessed the similarity between the two data clusterings using the Adjusted Rand index (ARI) and clustering entropy index. High ARI values and low entropy values are obtained across a wide range of clustering resolutions, apart from the initial resolution of 0.2 (Figures S4A and S4C). In addition, analysis of the clustering tree shows that at a resolution of 0.2, there are 11 clusters which continue to be split up gradually. At increasing resolutions, the number of in-proportion edges (edges with low transparency) remain low which indicates only minor changes in the clustering tree. At a clustering resolution of 0.8, we obtained 22 clusters (Figure S4B). When we re-examined the distribution of our tailbud labels against Wagner et al.’s labels, we find that they are highly concordant (Figure S3B), suggesting that our selection of the zebrafish tailbud cells are robust across different analytical strategies. As the Louvain algorithm is stochastic, we re-ran the algorithm for 10 iterations and retained cells that are consistently located in the tailbud clusters for 9 and 10 iterations for downstream analyses (Figure S4D).

##### Critical index and transcriptional noise index

The critical index is defined as the ratio of two averaged Pearson correlation coefficients: the average correlations between all pairs of gene vectors over the average correlations between all pairs of cell state vectors (Mojtahedi et al., 2016). In the scRNA-seq analysis, to account for the differences in cell number between clusters, 200 cells from each cluster were randomly sampled with replacement to calculate the index, and the procedure was repeated for 10,000 times. We also assessed the robustness of the critical index to differences in cell number and number of marker genes used (Figure S5).

The transcriptional noise index was measured using the top 2000 highly variable genes of each cluster following the work of Mohammed et al. (Mohammed et al., 2017).

#### Tailbud image registration with ZebReg

##### Overview of pipeline

ZebReg is a 3D, non-landmark-based image registration Python tool which we developed to integrate cellular position and nuclear gene-expression information from confocal images of zebrafish tailbuds. Leveraging on the open-source open3D library (Zhou et al., 2018), ZebReg implements a set of rigid body, point-based registration algorithms that are popular in the field of geometric registration to align a 3D point cloud (source cloud) into a reference point cloud (target cloud).

At present, we have tested ZebReg on zebrafish tailbuds ranging from 18ss to 30ss. Briefly, confocal images were first preprocessed in Imaris to obtain segmented DAPI-stained nuclei (Figure S1). Next, to ensure a consistent field of view, all nuclei posterior to the tip of the developing notochord for all the images were retained for analysis. ZebReg performs the alignment by first importing the 3D centroid coordinates and gene expression intensities (if present) of the segmented nuclei and converting each image into a point cloud. Then, given a set of source clouds and a reference point cloud (target cloud), ZebReg finds the best linear transformation (no shearing, stretching or other deformations) between each source cloud and the target cloud. In addition, if color intensities of the source clouds are provided, ZebReg can map them onto the target cloud by imputing the gene expression intensities in the target point cloud and thus generate a composite image (See Figure S7).

##### Imputation of gene expression intensities

There are three possible sets of outcomes during the imputation procedure:

i) First, the mapping of the source point to the target point may be unique, in which case the target point simply adopts the intensity value of the corresponding source point.
ii) In cases where there is not a single source point corresponding to the target point, ZebReg provides the user with several options to resolve the discrepancy. If multiple source points map to the same target point, the target point adopts either the mean or median of these source intensity values (default: ‘median’).
iii) Alternatively, if there is no source point that corresponds to the target point, ZebReg provides three options to impute the gene expression intensity of this target point: ‘null’, ‘complete’ or ‘knn’ (default ‘knn’). ‘null’ sets the intensity of the target point to 0, whilst ‘complete’ can be used if the target point cloud already has an intensity channel for that gene, in which case the point simply retains the original target intensity value. In the default ‘knn’ case, regression is performed based on the k-nearest neighbors of the point (default n = 5) as implemented in the *sklearn* package. The target point takes on the mean intensity value of the closest target points.

Notably, in cases ii) and iii), ZebReg imputes the expression intensities of the target points by borrowing information from multiple source or neighboring target points.

##### Point set registration algorithms

To conduct the image alignment, ZebReg employs the following point set registration algorithms:

i) Random Sample Consensus (RANSAC)

The RANSAC algorithm is a non-deterministic global alignment algorithm that is used in ZebReg to provide the initial coarse alignment for the ICP and cICP local algorithms (Fischler and Bolles, 1981).

ii) Iterative Closest Point (ICP)

In the vanilla ICP algorithm, the algorithm repeatedly updates the transformation required to map the source to target cloud by minimizing the distance between points (Besl and McKay, 1992). In ZebReg, we use the point-to-plane ICP variant due to its increased speed of convergence (Rusinkiewicz and Levoy, 2001).

iii) Colored Iterative Closest Point (cICP)

For images with a color channel in common, it is advantageous to consider their color on top of geometry during point set registration. In these cases, ZebReg uses cICP, a modified version of ICP implemented in open3D, which optimizes a joint geometric and photometric objective (Park et al., 2017).

ZebReg carries out all alignments by first performing a coarse global alignment with RANSAC, followed by either a finer alignment with ICP (if no color channel is supplied) or cICP (if a common color channel is present in the source and target image).

##### In silico validation of ZebReg

First, we constructed a mean absolute error (MAE) metric which quantifies the average difference in normalized signal intensities of the shared color channel between the source and target image pair after image registration. To assess the accuracy of ZebReg’s image alignment with the cICP algorithm, we selected a source and target point cloud of the zebrafish tailbud and used the MAE as the test statistic in a permutation test which tests the following hypotheses:

H_0_: ZebReg cICP registration has no effect on the color intensity residuals between source and target clouds.

H_1_: ZebReg cICP registration reduces the color intensity residuals between source and target clouds.

In the permutation test, the sampling distribution under the null hypothesis was constructed by randomly rearranging the order of the target color array, and then calculating the MAE using the permutated and original target color arrays over 10,000 iterations. In effect, the null distribution provides the range of MAE estimates under the condition where ZebReg’s reported correspondence mapping between the source and target color arrays is random. The null distribution was then fit to a Gaussian distribution for the computation of the 95% confidence interval (Figure S9A).

Next, to assess the effectiveness of the various point cloud registration algorithms, we registered a point cloud with its rotated counterpart using 3 algorithms that are implemented in open3D: RANSAC, ICP and cICP, and assessed whether they can successfully recover the correspondence map. (Figure S9B)

We then compared the cICP’s performance across the different datasets. The datasets we have chosen for comparison were (Figures S9C and S9D):

• *Test sample*: Images of two separate zebrafish tailbuds at 18ss. Test sample exemplifies the performance of the algorithm on an actual use case in practice.
• *Lateral halves*: Images of two lateral halves of a single 18ss tailbud image. Because the point clouds do not overlap, any correspondence between the points in Lateral halves are spurious.
• *AP*: Images of the anterior and posterior ends of a single 18ss tailbud image. Like Lateral halves, any correspondence found between points in AP is spurious.

In the absence of ground truth data or an alternative image registration method, to achieve a better grasp of ZebReg’s performance, we benchmarked the registration results of these three datasets onto a noise calibration curve, which we obtained from registering noise-shifted versions of the source cloud onto its original copy.

Zero-mean Gaussian distributions with standard deviations ranging from 0 to 30 were sampled to construct an array of noise matrices. These noise matrices were added to the positions of the source clouds to generate an array of noise-shifted point clouds. Conceptually, each noise-shifted point cloud is an *in silico* analogue of a tailbud that differs from its idealized, identical twin in nuclei position by a prespecified level of noise. To construct the noise-calibration curve, all noise-shifted point clouds were registered against the original source cloud, which returned the values for the fitness, inlier RMSE and inlier MAE metrics. For the heavily noise-shifted point clouds, many points are classified as outliers and therefore, the inlier metrics overestimate the registration quality by omitting these points. To correct this, we scaled the inlier RMSE and inlier MAE metrics by the corresponding fitness and plotted the scaled inlier RMSE and scaled inlier MAE values instead. Comparing our results (Figures S9E and S9F), we conclude that ZebReg’s registration of Test Sample outperforms the Lateral halves and AP datasets and returns acceptable fitness, inlier RMSE and MAE scores in its alignment of the Test Sample dataset.

##### Validation of ZebReg against HCR data

To assess whether the imputation procedure alters the gene expression distributions, we constructed Q-Q plots of the original and imputed gene expression distributions of 12 genes at 18ss (Figure S10A). For the purposes of the comparison of Q-Q plots, we also analyzed the expression of four additional genes (wnt8a, thbs2, id3 and depdc7a) that were not used in constructing the composite maps.

We also assessed the extent to which ZebReg maintains the quantitative relationships between genes in the NMps by comparing the pairwise linear correlations of imputed genes with the original correlations from the HCR datasets (Figure S10B). A total of 17 gene pairs were compared. As a measure of how close the original and imputed correlations are to each other, we computed the minimum difference between the imputed correlation and the associated original correlations (Figure S10C). The minimum difference was computed by calculating the differences between the correlations obtained from the HCR images and the correlation from the composite map, and then taking the minimum value of the differences.

##### Construction of in silico composite maps

To construct the composite maps for each stage, we first selected images across different samples to be used for the imputation. Each image consists of *sox2* and *tbxta* stained alongside one or two additional genes and belongs to an image group. Specifically, there are a total of five image groups that correspond to particular HCR experiments (Table S2): STT (*sox2, tbxta, tcf*), STHC (*sox2, tbxta, hes6, cdh6*), STSC (*sox2, tbxta, sp5l, cdh6*), STTC (*sox2, tbxta, tagln3b, cdh6*) and STZC (*sox2, tbxta, znf703, cdh6*). For each of the three composite maps (18ss, 24ss, 28ss), five images of the same stage, one from each of the five image groups, were mapped onto a chosen target image using sox2 as the common color channel for cICP alignment (Figure S8). These six images for each composite map were chosen to best reflect the number and spatial distributions of the *in silico* NMps in the resultant composite maps. In summary, each composite map combines information across six images (one for the target image and five for the source images) to generate an eight-dimensional (*sox2, tbxta, cdh6, hes6, sp5l, tagln3b, tcf, znf703*) point cloud image.

#### Analysis of the composite maps

##### Identification of in silico NMps

Following the construction of the composite map with ZebReg, imputed intensity values of all genes below the 0.7 quantile threshold were set to 0 and rescaled by min-max normalization (Figure S12A). Amongst the *sox2*+*tbxta*+ cells that were identified in the composite map, most were found within the approximate NMp spatial regions (Figures S12Bi and S12Bii). Of the *sox2*+*tbxta*+ points that reside outside of the NMp regions and are thus excluded from being NMps, they fall into two groups (Figure S12Bii). The first group corresponds to the hypochord cells that constitute the bulk of these *sox2*+*tbxta*+ non-NMps in the 18ss (47/146: 32%), 24ss (43/203: 21%) and 28ss (29/56: 52%) composite maps. The second group of cells are found in the 18ss composite map only (17/146: 11%) and are a small population of aberrant cells that have likely arisen from technical errors in either ZebReg’s alignment or mapping procedure. These cells flank the hypochord and floor plate and thus may have been mistakenly assigned above-background levels of *sox2*due to their proximity to these two *sox2*-expressing structures. After the removal of these two groups of cells, the resultant 78, 183 and 27 *in silico sox2*+*tbxta*+ cells in the 18ss, 24ss and 28ss composite maps are defined as the *in silico* composite map NMps. (Figure S12Biii)

##### Neural-mesodermal (NM) index construction

The neural-mesodermal index, $NM_{j}$ for the $j^{th}$ cell is defined as:

(Equation 1) $$NM_{j}=N_{j}-M_{j}$$

where $NM_{j}$, $N_{j}$, $M_{j}$ are the neural-mesodermal index, neural index and mesodermal index of the $j^{th}$ cell, respectively, and j=1,2,…,C for a total of C NMps.

The neural index, $N_{j}$, for the $j^{th}$ cell is defined as:

(Equation 2) $$N_{j}=sox2_{j}+\overset{G}{\underset{k=1}{\sum }}\tilde{{\text{ρ}}_{k}}\left(1-\varepsilon_{k}\right)Gene_{kj}$$

where $Gene_{kj}$ is the min-max normalized expression intensity of the $k^{th}$gene in the #$j^{th}$ cell; $\tilde{{\text{ρ}}_{k}}$ is the median of the Pearson’s correlation coefficients of $Gene_{k}$ and *sox2*, computed from the NMps segmented from all the HCR images of the same somite stage; $\varepsilon_{k}$ is the interquartile range of $Gene_{k}$’s correlation coefficients. The ($1-\varepsilon_{k}$) term penalizes $Gene_{k}$’s contribution to the neural index if it displays large variability in its correlation coefficients between all the HCR images of that somite stage. The summation is applied to all the genes, G, minus *tbxta* and *sox2*. The total number of genes is G+2.

The mesodermal index, M, is defined symmetrically but with *tbxta* replacing *sox2* and the correlation coefficients calculated with respect to *tbxta* instead. We further verified that the NM index provides a sensible summary of the NMp’s neural/mesodermal potential (Figures S13B–S13D) and is not systematically biased towards either neural or mesodermal indices (Figures S13E and S13E′).

##### Construction of the NMp probability map

Tailbud images (source images) were aligned to an arbitrarily chosen target image tailbud. The NMp nuclei in the source images were pre-segmented prior to alignment in Imaris (Bitplane) and hence, it is possible to keep track of the number of times each target cell receives a mapping from a source NMp cell. Target cells with a large count number is assigned a high probability of being an NMp. For visualization purposes, in my probability maps, we displayed only target cells with a minimum count number of 2 for each probability map (Figure S14).

##### Standard error of empirical entropy estimation

The standard error was estimated by the leave-one jackknife resampling method and is implemented using the R *bootstrap* package (Efron and Tibshirani, 1993; Wiesner et al., 2017). In this method, the entropy was repeatedly estimated but with one of the data points randomly removed during each computation.

### Quantification and statistical analysis

Levene’s test for the equality of variance was carried out for the NMp numbers at 24ss (Figure 1) against the other four timepoints. ∗p-value < 0.01. For all boxplots (Figure 2), the lower and upper hinges correspond to the first and third quartiles. In addition, the upper whisker extends from the hinge to the largest value no further than 1.5 times the interquartile range. Outlier samples are colored in red. Wilcoxon-Mann-Whitney unpaired two-sample test ∗∗∗∗p-value < 0.0001; ns = not significant.

## Data Availability

• This article analyzes existing, publicly available RNA seq data. The accession numberfor the dataset is listed in the key resources table. Microscopy data reported in this article will be shared by the lead contact on request. • All original code has been deposited at Zenodo and is publicly available as of the date of publication. The DOI is listed in the key resources table. • Any additional information required to reanalyze the data reported in this article is available from the lead contact on request.

## Supplemental information

Document S1. Figures S1–S14, Tables S1 and S2

Table S3 containing list of marker genes for cell clusters identified in Figure 2A
